# Chronic Disease Monitoring: Methodology for Classification Error and Self-Selection Bias Correction in Clinical Laboratory Data

**DOI:** 10.3390/healthcare13233056

**Published:** 2025-11-25

**Authors:** Jesuan Betancourt, Efrain Betancourt, Abiel Roche-Lima, Julian Velev

**Affiliations:** 1 Abartys Health, San Juan, PR 00907-3913, USA; jbetancourt@abartyshealth.com (J.B.); ebetancourt@abartyshealth.com (E.B.); 2 Center for Collaborative Research in Health Disparities, RCMI Program, Medical Science Campus, University of Puerto Rico, San Juan, PR 00936-5067, USA; abiel.roche@upr.edu; 3 Department of Physics, University of Puerto Rico, San Juan, PR 00925-2537, USA

**Keywords:** public health, chronic disease monitoring, diabetes mellitus

## Abstract

**Background/Objectives**: Chronic diseases are among the leading causes of morbidity and healthcare costs worldwide. Diabetes mellitus is one of the most prevalent and costly chronic conditions in the United States, with a disproportionate burden in Puerto Rico. Surveillance of diabetes relies mainly on infrequent cohort studies and self-report surveys, which are limited in accuracy, segmentation, and timeliness. This study aimed to develop a generalizable methodology for monitoring chronic disease prevalence using routinely collected laboratory data, while correcting for systematic biases and diagnostic errors. **Methods**: We analyzed more than five years of de-identified laboratory test results (2020–2024) from a large, island-wide network of clinical laboratories in Puerto Rico. To produce unbiased prevalence estimates, we applied a mathematical correction framework that accounted for two main sources of distortion: (1) classification errors from treatment effects and test limitations, quantified through confusion matrices derived from longitudinal records; and (2) self-selection bias from differential testing rates, estimated empirically by demographic segment. Demographic reweighting ensured representativeness with respect to census data. **Results**: Using diabetes as a test case, corrected estimates for 2024 showed an adult prevalence of 18.0%, compared to 14.1% based on raw laboratory frequencies. The large amount of data provided high-resolution estimates by age, sex, and location, enabling fine-grained detection of demographic and geographic disparities. **Conclusions**: Bias-corrected laboratory surveillance provides accurate, timely, and demographically representative estimates of chronic disease prevalence. The methodology is scalable, cost-effective, and broadly applicable to other multi-stage chronic conditions, offering a foundation for next-generation public health monitoring and targeted interventions.

## 1. Introduction

Diabetes mellitus is one of the most prevalent and costly chronic conditions in the United States [1]. According to the Centers for Disease Control and Prevention (CDC), the prevalence of diagnosed diabetes among adults has risen steadily—from 9.5% in 2000 to 14.8% in 2022—with an additional 38% classified as prediabetic [2]. The economic burden is considerable, with annual costs exceeding $337 billion in direct medical spending and lost productivity [3]. In addition to its scale, the impact of diabetes is unevenly distributed across the population, with significant disparities by income, education, and ethnicity. The geographical prevalence distribution shows much higher prevalence rates in the Southern states and Puerto Rico (PR) than in the rest of the country [2,4], which incidentally are the lowest-income areas in the United States [5].

Given the scale and impact of diabetes, one might expect a robust nationwide infrastructure for monitoring its prevalence and progression. In practice, however, chronic disease surveillance in the United States remains limited, relying heavily on periodical surveys and physical examinations conducted on small cohorts. The primary source of national diabetes prevalence data is the National Health and Nutrition Examination Survey (NHANES) [6], a biennial cohort study that combines interviews with clinical measurements to assess health trends in the U.S. population. Despite its careful design, NHANES includes only around 15,000 participants per cycle—translating to fewer than 1000 individuals per demographic subgroup—and is updated only every two years. Although more rigorous than simple self-report surveys, NHANES remains fundamentally retrospective, infrequent, and limited in resolution.

In PR, the situation is even more problematic. NHANES does not cover the territory, and the sole source of prevalence data is the CDC’s Behavioral Risk Factor Surveillance System (BRFSS), a phone-based self-report survey [7]. According to BRFSS in 2021, 14.8% of respondents reported having been diagnosed with diabetes, and just 4.3% reported being prediabetic. A major limitation of phone-based surveys is that they are prone to strong response bias. Individuals who answer unsolicited calls are not representative of the general population, and social desirability bias may lead respondents to withhold or underreport sensitive health information–especially when survey anonymity is uncertain.

Academic studies incorporating both interviews and physical exams are not very common in PR [8,9,10], but the most recent comprehensive investigation found that 25.5% of adults met the diagnostic criteria for diabetes and 47.4% for prediabetes [11]. These findings confirm the notion that the prevalence of diabetes is underreported in phone interviews and that most people are unaware that they are at high risk for diabetes.

While traditional survey methods fall short in timeliness and accuracy, electronic health records (EHRs) have emerged as a potential alternative for chronic disease surveillance [12]. In response to the Health Information Technology for Economic and Clinical Health (HITECH) Act, several monitoring initiatives have been proposed that leverage EHR data–both structured (e.g., ICD-10 codes) and unstructured (e.g., laboratory results–to track disease prevalence and incidence [13,14,15,16,17]. However, EHR-based monitoring systems face significant challenges. EHRs are primarily designed for patient-level documentation and billing, not for population-scale analytics. The ecosystem is highly fragmented, with heterogeneous software systems and variable data quality across providers. Lack of standardization, misclassification, missing data, and difficulties in linking patient records across institutions make large-scale aggregation and interpretation problematic [18,19,20].

In contrast to EHR-based systems, our approach leverages data from Laboratory Information Systems (LIS)—specialized providers that collect, process, and aggregate clinical laboratory test results. However, as with all observational health data, the use of LIS data for prevalence estimation must contend with important sources of bias. Chief among these is *self-selection bias*: individuals do not enter the tested population randomly but rather based on a combination of clinical and behavioral factors. People with symptoms or known diagnoses are more likely to be tested, but so are those in specific demographic groups–such as older adults, females, or individuals more engaged with the healthcare system. As a result, the tested population systematically differs from the general population. A second source of error is *classification errors*, which arises from both the intrinsic limitations of diagnostic tests (e.g., sensitivity and specificity) and from treatment effects—such as the use of medications that artificially suppress biomarker values and obscure disease status [21,22,23].

It is worth noting that EHR-based monitoring systems are also subject to self-selection bias [13,14,15,16]. And laboratory results used by these systems are prone to classification error, as illustrated by the 87% sensitivity of hemoglobin A1c-based diabetes diagnoses when compared to ICD-10 coding [23]. While some studies attempt to validate laboratory-based prevalence estimates against cohort data [13,15,24], these comparisons are of limited value given the small size of most cohort studies and the absence of any systematic procedure for correcting the underlying observational biases.

In this study, we introduce a novel methodology for real-time monitoring of chronic disease prevalence using LIS-derived laboratory data. Our approach explicitly corrects for self-selection bias and classification errors through a rigorous mathematical framework that relies on only a few empirically derived parameters. These parameters are estimated using more than five years of testing data collected from a large, island-wide network of clinical laboratories in PR, encompassing millions of test results.

To demonstrate its utility, we apply the framework to diabetes mellitus, where it yields accurate, demographically representative estimates of both diabetes and prediabetes prevalence. Importantly, the methodology is generalizable: it can be extended to other multi-stage chronic conditions that are routinely monitored through laboratory testing. By enabling accurate, low-cost, and continuously updated disease surveillance, our approach provides a scalable foundation for next-generation public health infrastructure–integrating big-data analytics, epidemiological modeling, and timely decision support.

## 2. Materials and Methods

### 2.1. Data

**Figure 1:**
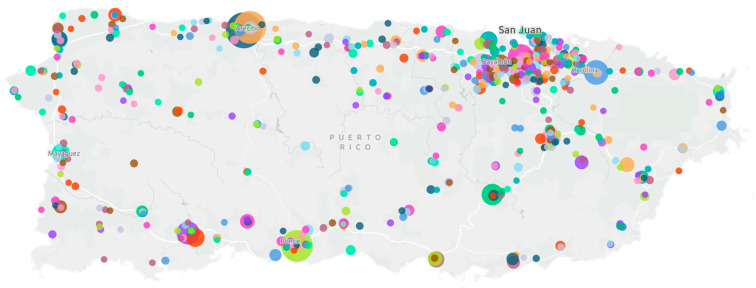
Geographic distribution and relative test volume of clinical laboratory sites across Puerto Rico. Each laboratory is assigned a randomly generated color to enhance individual site visibility.

Data for this study were obtained from the clinical results datalake of Abartys Health (San Juan, PR, USA), which aggregates laboratory test results from the two major laboratory information system providers in PR. The dataset incudes results from approximately 800 clinical laboratory sites—representing the majority of laboratories on the island—and is broadly distributed across all municipalities, ensuring wide geographic coverage. The spatial distribution of laboratory locations and their relative testing volumes is shown in Figure 1, demonstrating comprehensive access to routine testing across the jurisdiction.

All records were de-identified by removing personally identifiable information (e.g., names, dates of birth, addresses), and unique patients were assigned non-descriptive identifiers. Demographic variables available in the dataset included age, sex, and location (zip code or census tract). Geocoding was performed prior to de-identification to determine the census tract. Additional contextual information such as ordering physician and insurance provider was also captured, while data on ethnicity, income, education, and family history were not available. Each test was timestamped, labeled with its corresponding Logical Observation Identifiers Names and Codes (LOINC) [25], and standardized across units. In accordance with US CFR 46.104(d), secondary analysis of de-identified data does not require patient consent. The study protocol was reviewed and approved by the University of Puerto Rico–Medical Sciences IRB (Ref. 2301072914).

Our analysis focuses on the most recent complete calendar year available in the dataset, which defines the period of interest (POI). Diabetes diagnosis is typically based on blood glucose measurements, including fasting plasma glucose (FPG), random (non-fasting) plasma glucose, and oral glucose tolerance tests (OGTTs) [26]. While OGTTs are considered the clinical gold standard, they are rarely used in practice due to their time and resource requirements.

For population-level surveillance, FPG is particularly well suited, as it is widely administered as part of routine comprehensive metabolic panels, reducing selection bias. In contrast, hemoglobin A1c (A1C) —which reflects average glucose levels over several months—is more commonly ordered when diabetes is already suspected or being managed, making it more susceptible to indication bias. To preserve population representativeness, we excluded tests ordered primarily for diagnostic confirmation or clinical monitoring (e.g., A1C and OGTT), omitted specialized laboratory settings such as hospital-based labs, and restricted the analysis to adults aged ≥20 years. The assumptions and conditions for unbiased estimation from observational laboratory data are discussed in greater detail in the Supplementary Materials.

To enhance consistency and reduce the noise introduced by high-frequency testing—especially during acute interventions, we aggregated individual-level test results into monthly intervals. Glycemic values were grouped and averaged within each interval to provide a meaningful snapshot of each individual’s longer-term glycemic state. This approach balances temporal resolution with the need for robust, interpretable indicators of chronic health conditions. Fasting status was controlled by laboratories and used as reported.

Table 1 summarizes the test volume and patient demographics for the POI, while Supplementary Figure S1 details the distribution of tests by sex and age group. The longitudinal dataset, spanning 2020–2024, was used to estimate the empirical parameters for correcting classification errors and self-selection bias. This dataset, comprising millions of glycemic test results across PR, is summarized in Supplementary Table S1.

### 2.2. Methods

The detailed methodology used to estimate unbiased prevalence for multi-stage chronic conditions is presented in the Supplementary Materials. Here we treat diabetes as a 3-stage disease, where $D_{0}$ denotes normoglycemic (healthy) individuals, $D_{1}$ prediabetic, and $D_{2}$ diabetic, classified in accordance with the American Diabetes Association (ADA) fasting plasma glucose thresholds [26]. These thresholds capture all-diabetes prevalence (type 1 + type 2), as clinical differentiation between subtypes was not attempted in this stage. To estimate the true population prevalence vector $P=\left(P_{D_{0}},P_{D_{1}},P_{D_{2}}\right)$, we begin with the observed positive frequencies in the sample $F=\left(F_{D_{0}},F_{D_{1}},F_{D_{2}}\right)$ and apply a stepwise correction process that accounts for bias and error (illustrated in Supplementary Figure S3).

#### 2.2.1. Demographic Reweighting

To ensure that the overall prevalence estimate is representative of the population, we stratified the sample into demographic segments $S_{l}$, where each segment or stratum corresponds to a unique combination of parameters such as sex and age. The true population weight of each stratum ($\omega_{l}$) was obtained from U.S. Census data [27], while the corresponding weight in the laboratory sample was denoted $\omega_{l}^{S}$.

We compute prevalence estimates within each demographic segment independently. These segment-level estimates are then aggregated into a population-level estimate using a weighted sum

$$P\left(D_{i}\right)=\sum_{l}\omega_{l}\cdot P\left(D_{i}|S_{l}\right)$$

where $P\left(D_{i}|S_{l}\right)$ is the prevalence of stage $D_{i}$ within segment $S_{l}$ and $\omega_{l}$ is the weight of segment $S_{l}$ in the general population. This step ensures that the final prevalence estimate is demographically representative of the total population.

To support statistical stability at the level of demographic and geographic strata, analyses were conducted only in strata with several thousand observations, which yields sampling variability below approximately one percentage point for typical chronic disease prevalence ranges. This criterion ensures robust estimation even when stratifying the population into multiple demographic groups.

#### 2.2.2. Classification Errors

Even when tests are used consistently, no diagnostic method is perfectly accurate. In the case of diabetes, classification errors arise not only from intrinsic limitations of the FPG test itself but also from the effects of medication—particularly insulin—on blood glucose levels. These medications can mask the true disease state by lowering glycemic values, resulting in diabetic individuals appearing prediabetic or even normoglycemic in lab results.

While confusion matrices are typically used to correct for the intrinsic sensitivity and specificity of a diagnostic test (e.g., FPG)—by comparing its results to a gold-standard reference (e.g., OGTT)—such validation data are not available at population scale in this context. Consequently, intrinsic test performance has not been estimated.

In our framework, the confusion matrix serves a different purpose: it captures transitions between observed glycemic states driven primarily by medication use and other clinical interventions. Individuals who have ever met the diagnostic criteria for diabetes ($D_{2}$) are treated as members of the latent diabetic group for the remainder of the observation window. Improvements in glycemia due to pharmacologic therapy, lifestyle change, or other treatments are therefore represented as observed-state transitions $\left(D_{2}\to \{D_{1}^{*},D_{0}^{*}\}\right)$, reflecting remission in laboratory values without implying full reversal of the underlying metabolic disease state.

Using five years of historical data (2020–2024), we examined follow-up A1C test results for individuals previously classified as diabetic to estimate the likelihood of being observed in each apparent glycemic category—normoglycemic ($D_{0}^{*}$), prediabetic ($D_{1}^{*}$), and diabetic ($D_{2}^{*}$). This yielded empirical estimates for the corresponding elements of the confusion matrix K, defined as

$$K_{D_{i}^{*}D_{2}}=P\left(D_{i}^{*}|D_{2}\cap S\right)$$

where S denotes the laboratory population. Further details on the estimation of the confusion matrix are provided in the Supplementary Materials.

Once the confusion matrix K is known, we correct the observed positive frequencies for classification error. The classification-error-adjusted frequency vector $\bar{F}$ is computed as

$$\bar{F}=K^{-1}\cdot F$$

where F is the raw observed frequency vector and $K^{-1}$ is the inverse of the empirical confusion matrix. Because K is triangular with unit diagonal in the $D_{i}$/$D_{1}$ block, the only invertibility condition is $K_{22}>0$, which held in all strata. This transformation can be viewed geometrically as a rotation in the space of classification labels, aligning the observed categories $D_{i}^{*}$ with their true counterparts $D_{i}$, as conceptually illustrated in Supplementary Figure S2.

Correcting for classification errors ensures that downstream prevalence estimates are not skewed by the effects of glycemic control therapies, particularly in populations with high rates of insulin usage.

#### 2.2.3. Self-Selection Bias

Clinical laboratory data does not constitute a random sample of the general population. Instead, individuals self-select into testing based on a combination of demographic and health-related factors. For example, older adults and females are more likely to be tested due to age-related conditions, preventive care practices, and sex-specific patterns of healthcare engagement. Simultaneously, individuals experiencing symptoms or previously diagnosed with a condition are more likely to be tested and tested more frequently.

In the case of diabetes, this results in an overrepresentation of prediabetic ($D_{1}$) and diabetic ($D_{2}$) individuals relative to normoglycemic individuals ($D_{0}$), which, if uncorrected, leads to inflated estimates of prevalence. Together, these factors give rise to *self-selection bias*, wherein the laboratory-tested sample is skewed toward individuals who differ systematically from the general population in both demographic composition and disease risk.

To adjust for this bias, we estimate the stage-specific testing rates $B_{D_{i}}$, which represent the probability that an individual at stage $D_{i}$ was tested within the POI. These rates are derived empirically from the longitudinal dataset spanning 2020–2024. For each individual, we construct a time series of FPG test results and determine their condition stage. We then compute the average testing interval $\tau_{i}$ for individuals in each stage $D_{i}$, and estimate the probability of being tested in the POI as

$$B_{D_{i}}=P\left(S|D_{i}\cap S_{l}\right)\approx P\left(\tau \leq T|D_{i}\cap S_{l}\right)$$

where S denotes inclusion in the sample during the POI of duration T, and $S_{l}$ represents a specific demographic segment (e.g., age and sex). Further details of the estimation procedure are provided in the Supplementary Materials.

Incorporating these testing rates into the estimation framework, we adjust the classification-error-corrected frequencies $\bar{F}$ by solving the following system of equations

$$\sum_{j}\left({\bar{F}}_{D_{i}^{*}}-\delta_{ij}\right)B_{D_{j}}P_{j}=0$$

subject to the constraint $\left|P\right|=1$, where ${\bar{F}}_{D_{i}^{*}}$ are the classification-error-corrected frequencies, $\delta_{ij}$ is the Kronecker delta, $B_{D_{j}}$ are the stage-specific testing rates, and $P_{j}$ are the unknown unbiased prevalence values to be estimated. Solving this system yields the final unbiased prevalence estimator $P=\left(P_{D_{0}},P_{D_{1}},P_{D_{2}}\right)$, accounting for both classification error and self-selection sampling bias. When the self-selection term is set to unity (i.e., no differential testing propensity), the expression reduces to the Rogan-Gladen estimator [22] as shown in the Supplementary Materials, providing a direct link to established methods and validating the internal consistency of the approach.

## 3. Results

We demonstrate the methodology by constructing a chronic disease monitoring system, using diabetes as a test case. In this application, Individuals are classified as prediabetic if their FPG levels fall between 100 and 126 mg/dL, and as diabetic if FPG exceeds 126 mg/dL as established by ADA [26]. We rely on FPG alone for prevalence estimation, as it is routinely administered as part of comprehensive metabolic panels [28], making it less susceptible to selection bias than A1C tests. This example illustrates how the framework can be applied to other chronic conditions with well-defined biomarker thresholds and standardized laboratory tests.

### 3.1. Bias and Error Correction

**Figure 2:**
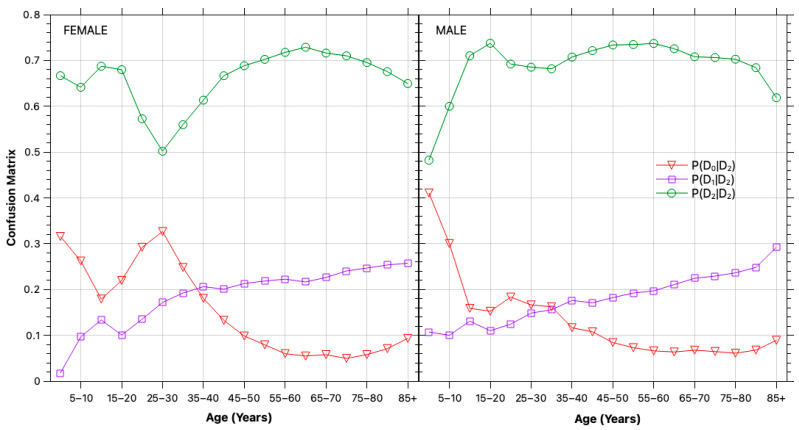
Classification error. Estimated elements of the confusion matrix showing the fraction of previously diagnosed diabetic individuals ($D_{2}$) observed as normoglycemic ($D_{0}^{*}$), prediabetic ($D_{1}^{*}$), or diabetic ($D_{2}^{*}$) during 2020–2024.

The estimated confusion matrix elements—i.e., the fraction of individuals with a known diabetes history testing as diabetic, prediabetic, or normoglycemic—are shown in Figure 2 for the different population segments. The results indicate that while the majority continue to test within the diabetic range, a significant portion (~20%) appears prediabetic, and a smaller fraction (~5%) test within the normal range, reflecting glycemic control through insulin or other interventions.

**Figure 3:**
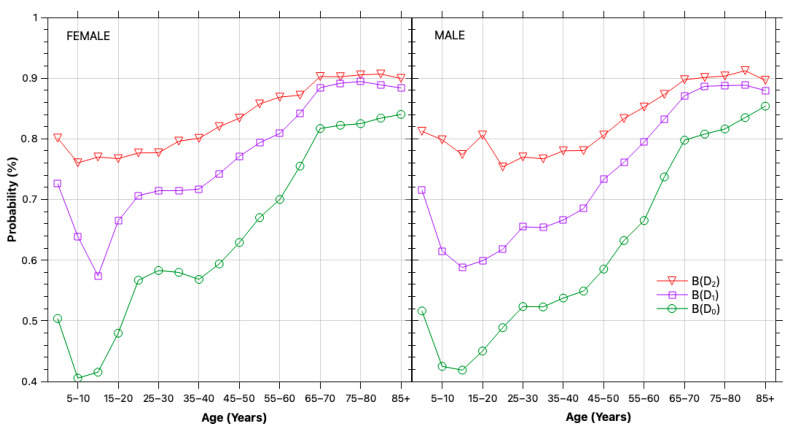
Self-selection bias. Estimated probability of undergoing an FPG test within the period of interest (2024), stratified by disease stage—normoglycemic ($D_{0}$), prediabetic ($D_{1}$), and diabetic ($D_{2}$) —and by demographic segment. Based on longitudinal data from 2020–2024.

To estimate self-selection bias, we used the historical dataset to count the number of tests performed during the POI for individuals in each population segment and disease stage, and from these counts derived the corresponding testing frequencies. The stage-specific testing probabilities are shown in Figure 3, which demonstrates that individuals with diabetes are tested much more frequently than healthy individuals, particularly in younger and middle-aged cohorts. Although the gap narrows with age—as older individuals are more likely to be tested—the bias remains substantial and must be corrected.

### 3.2. Prevalence Estimation

**Figure 4:**
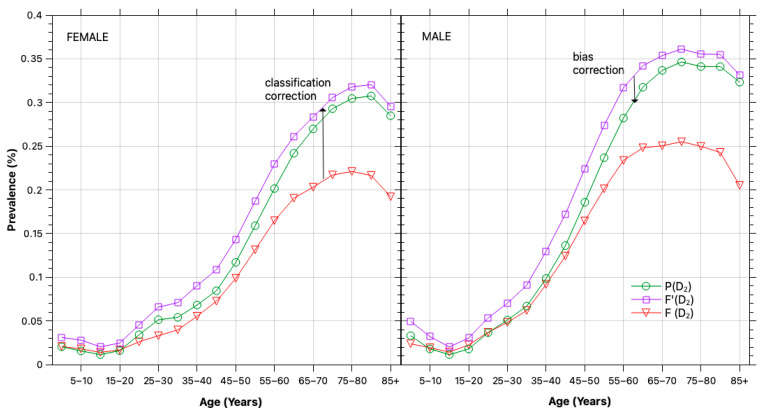
Diabetes prevalence. Corrected prevalence ($P_{D_{i}}$, green) by sex and age segment in 2024, shown alongside the raw positive frequencies ($F_{D_{i}}$, red) and the classification-error-adjusted frequencies (${\bar{F}}_{D_{i}}$, purple) for comparison.

By applying the full sequence of bias and error corrections, we obtain unbiased estimates for the population-level prevalence of diabetes and prediabetes in PR. Figure 4 illustrates the segment-level diabetes prevalence and the impact of bias and error corrections. The raw positive frequencies ($F_{D_{i}}$, red lines) significantly underestimate diabetes prevalence due to classification errors, particularly among individuals using insulin or other glucose-lowering medications.

At the same time, self-selection bias inflates prevalence estimates by overrepresenting individuals who are more likely to be tested. These two biases act in opposite directions: classification error suppresses observed prevalence, while self-selection bias exaggerates it. As seen in the comparison between the raw frequencies and the final corrected estimates ($P_{D_{i}}$, green lines), the net effect varies by segment but results in substantial misestimation if left uncorrected.

A complementary dynamic is observed for prediabetes in Supplementary Figure S4. Raw frequencies substantially overestimate prevalence due to diabetic individuals appearing as prediabetic due to effective glycemic control. Classification error correction reassigns these misclassified cases to their true category, and subsequent adjustment for testing bias adds to the effect. The combined corrections yield more balanced estimates across demographic segments.

**Figure 5:**
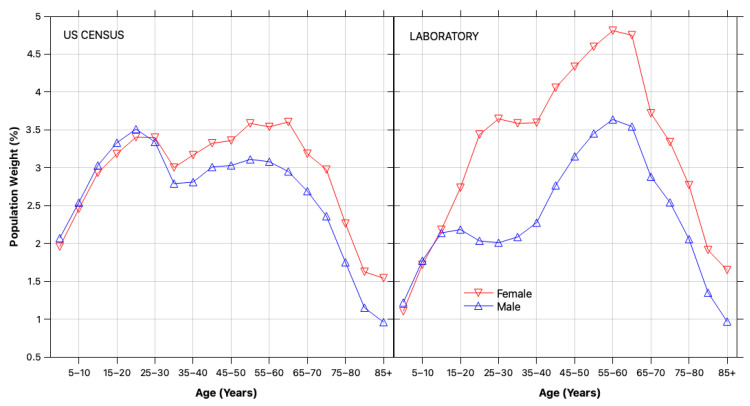
Demographic reweighting. Relative weights of population segments defined by sex and age group in the laboratory-tested sample ($\omega_{l}^{S}$, **left**) and in the general population ($\omega_{l}$, **right**), based on U.S. Census data for 2024.

These corrections are most pronounced in older cohorts, where both testing frequency and medication use are elevated. Without adjustment, segment-level estimates can be misleading, and population-level prevalence becomes inflated due to the demographic skew of the tested sample. Figure 5 illustrates this mismatch, highlighting the overrepresentation of older adults and females in the laboratory-tested population. The laboratory sample represents approximately 59% of PR’s total population of 3.286 million. To correct for this imbalance, demographic reweighting was applied using segment weights derived from U.S. Census data, as detailed in the Supplement.

Finally, the overall prevalence estimates for adults (≥20 years of age) across both sexes is shown in Table 2, alongside the raw positive frequencies $F_{D_{i}}^{S}$ observed in the laboratory sample. These corrected estimates show a substantially higher true diabetes prevalence compared to unadjusted values. The corrected overall diabetes prevalence for 2024 is estimated at 18.0%, compared to a raw frequency of 14.1%. Prediabetes prevalence is adjusted downward—from 28.9% in the raw data to a corrected estimate of 22.8%. Longitudinal trends in prevalence across the full historical period (2020–2024) are provided in the Supplementary Table S2.

These results confirm that diabetes and prediabetes remain major public health concerns in PR, with prevalence rates that exceed U.S. national averages.

## 4. Discussion

In this study, we developed and implemented an error- and bias-corrected methodology for estimating the prevalence of multi-stage chronic conditions using observational laboratory data. Leveraging this methodology and more than five years of glycemic test data from PR, we demonstrated the *first diabetes monitoring system* on the island.

### 4.1. Demographic Trends

The corrected prevalence estimates reveal several consistent patterns across demographic segments. Most notably, both diabetes and prediabetes prevalence increase steadily with age, peaking in older adults before declining slightly in the oldest cohorts. This late-life dip is likely due to selective mortality—individuals with more advanced disease may be underrepresented in the oldest age groups due to higher mortality rates [29].

We also observe a persistent sex-based disparity: males exhibit higher prevalence rates than females across nearly all age brackets. This trend remains after correction for sampling and classification biases and is likely reflective of a combination of biological, behavioral, and socioeconomic factors. Tellingly, while women are more frequently tested, men consistently show worse glycemic outcomes.

These findings underscore the importance of applying demographic corrections when using observational lab data for surveillance. Without proper reweighting, estimates would be skewed toward the health profiles of more frequently tested groups, obscuring the true distribution of disease across the population.

### 4.2. Geographic and Socioeconomic Trends

**Figure 6:**
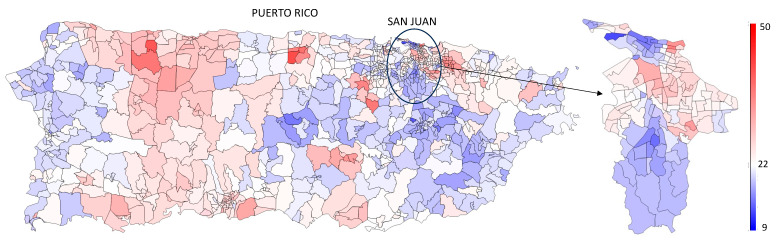
Diabetes prevalence in PR by census tract. High-resolution map of diabetes prevalence in 2024 estimated from laboratory data, aggregated at the census tract level. The inset shows a detailed view of the San Juan municipality, highlighting local variation across neighborhoods.

In addition to demographic variation, our analysis reveals substantial geographic and socioeconomic disparities in diabetes prevalence across PR. By geocoding patient addresses and aggregating results by census tract, we constructed a high-resolution map of diabetes prevalence, shown in Figure 6; the corresponding county-level distribution is provided in Supplementary Figure S5. This fine-grained spatial distribution reveals pronounced variability across neighborhoods, even within the same municipality. While some areas show relatively low prevalence, others—often just blocks away—exhibit disproportionately high rates.

The inset in Figure 6 provides a focused view of San Juan, highlighting this intra-municipal heterogeneity. For example, affluent neighborhoods such as Old San Juan and Condado exhibit much lower diabetes prevalence than nearby low-income communities like the public housing complex Llorens Torres, which is shown at larger scale in Supplementary Figure S6. Such high-resolution mapping is unthinkable by standard methods of for chronic disease surveillance.

To further explore the relationship between socioeconomic status and disease burden, we linked prevalence estimates at the municipality level to median household income data from the Social Determinants of Health (SDOH) database [30]. As shown in Supplementary Figure S7, there is a strong inverse relationship between income and diabetes prevalence. A linear regression analysis reveals that diabetes prevalence declines by approximately 2.9 percentage points for every $10,000 increase in income, with income alone explaining nearly a quarter of the observed geographic variation (*R*^2^ = 0.24). These findings agree well with other studies that diabetes prevalence was significantly higher in neighborhoods with lower median family incomes [23].

These results underscore the critical role of socioeconomic conditions in shaping chronic disease patterns. Importantly, they demonstrate the unique value of high-resolution laboratory-based surveillance in identifying localized health disparities that may be invisible in coarser, survey-based datasets.

### 4.3. Methodological Considerations

A key strength of our methodology is the clear separation between the *mathematical framework for prevalence correction*, which is exact, and the *empirical estimation of model parameters*, which necessarily introduces approximation. Although this initial implementation does not include a formal bootstrap or cross-validation procedure, the large sample size, near-universal testing coverage, and geographically distributed laboratory network provide empirical stability to the parameter estimates. Future work will explore resampling-based approaches to quantify uncertainty in these correction parameters.

The framework specifically addresses *classification error* and *self-selection bias*—two sources that, in principle, encompass most of the structural distortions present in observational health data, including differences in care-seeking behavior, demographic representation, and testing practices. A major contributor to classification error is the effect of pharmacologic therapy, which can lower glycemic values enough to produce an apparent remission in laboratory measurements. In most cases, such improvements reflect glycemic control rather than complete reversal of the underlying disease process. While some residual error may remain due to unmeasured factors or imperfect parameter estimation, these influences are expected to be small relative to the biases explicitly corrected. In addition, demographic stratification provides an added layer of robustness against population-level confounding.

The accuracy of the final estimates depends on the representativeness of the input data. To support this, the dataset must meet several criteria [20]. First, the laboratory network must provide widespread geographic coverage and include all major population strata. Second, the observation period must be sufficiently long to capture variation in health-seeking behavior. Third, the primary diagnostic test should be part of standard, broadly administered screening to minimize referral and indication bias. These requirements are described in more detail in the Supplement.

Although this study focuses on diabetes, the same bias-correction framework can be applied to other chronic conditions monitored through routinely ordered laboratory markers. Examples include creatinine-based estimation of kidney function (eGFR), lipid profiles for dyslipidemia, and thyroid function tests for hypothyroidism screening. These biomarkers are widely obtained in routine care and therefore meet the criteria for scalable population-level disease surveillance using this methodology.

Finally, although no independent population-level biomarker dataset currently exists in PR for direct validation, our prevalence estimates align with expected epidemiological patterns and with evidence from other settings demonstrating that biomarker-based surveillance consistently identifies a higher burden of undiagnosed diabetes than self-reported survey data. Future integration with electronic health records or claims databases would allow quantitative calibration of the correction framework once such data become accessible.

## 5. Conclusions

We presented a generalizable, bias-corrected methodology for estimating chronic disease prevalence in near-real-time using observational clinical laboratory data. Applied to diabetes in PR, the approach produced accurate, high-frequency, demographically and geographically resolved estimates by correcting for key sources of bias—including classification error and self-selection bias—using empirically derived parameters. The method is practical, scalable, and adaptable to other conditions, offering a cost-effective alternative to traditional surveillance systems. The enabling methodology readily extensible to other multi-stage chronic conditions provided appropriate biomarkers and longitudinal data are available. Our work highlights how the integration of big data and principled analytics can transform public health surveillance, contributing to reduced healthcare costs, more targeted interventions, and ultimately, improved population health and quality of life.

## Figures and Tables

**Table 1:** Sample size and glycemic test volume for adults (≥20 years) during the period of interest (2024).

Sex	Persons	Results	Fasting Plasma Glucose (FPG)	Hemoglobin A1c (A1C)
Female	433,641	1,065,532	744,054	321,478
Male	300,206	716,516	500,030	216,486
Both	733,847	1,782,048	1,244,084	537,964

**Table 2:** Population-level prevalence of diabetes and prediabetes. Corrected prevalence estimates for diabetes $(P_{D_{2}}$) and prediabetes ($P_{D_{1}}$) among adults and stratified by sex in 2024. Raw positive frequencies from the sample ($F_{D_{i}}^{S}$) are shown for comparison.

Sex	$P_{D_{2}}$ (%)	$F_{D_{2}}^{S}$ (%)	$P_{D_{1}}$ (%)	$F_{D_{1}}^{S}$ (%)
Female	16.3	12.5	19.9	25.7
Male	20.0	16.0	26.0	32.6
Both	18.0	14.1	22.8	28.9

## Data Availability

The data that support the findings of this study were licensed from Abartys Health for the purposes of this study alone and are not publicly available. Data access requests can be addressed to the corresponding author who would relay them to Abartys Health. Reasonable requests for access to the original code used to analyze the data can be directed to the corresponding author.

## Supplementary Materials

The following supporting information can be downloaded at: https://www.mdpi.com/article/10.3390/healthcare13233056/s1. Table S1: Sample size and glycemic test volume for the full dataset (2020–2024). Figure S1: Test counts by sex and age group in 2024. Number of tests performed per demographic segment for fasting plasma glucose (left panel) and hemoglobin A1c (right panel), stratified by sex and age group. Figure S2: Venn diagram illustrating the relationship between observed classifications in the tested sample and true disease stages in the general population, shown for a two-stage condition (left) and a multi-stage condition (right). Due to test imperfections, the observed groups ($D_{i}^{*}$) do not align with the true condition stages ($D_{i}$), leading to misclassification. Figure S3: Workflow for constructing the unbiased prevalence estimator. Starting from the observed positive frequencies $F_{D_{i}^{*}}$ for each observed disease stage $D_{i}^{*}$ and population stratum $S_{l}$, the unbiased estimator is built step by step using empirical parameters derived from historical data. $K_{D_{i}^{*}D_{j}}$ denotes the confusion matrix, which corrects for classification errors; $B_{D_{j}}$ represents the stage-specific testing frequency bias; and $\omega_{S_{l}}$ are the population weights for each stratum $S_{l}$. Figure S4: Prediabetes prevalence by sex and age group in Puerto Rico in 2024. Estimated prevalence of prediabetes across demographic segments, stratified by sex and age group. Results reflect corrected esti-mates after adjustment for classification error and self-selection bias. Table S2: Historical prevalence of diabetes and prediabetes in Puerto Rico (2020–2024). Yearly estimates of diabetes and prediabetes prevalence among adults, based on laboratory data corrected for classification error and self-selection bias. Figure S5: Diabetes prevalence in Puerto Rico by county in 2024. Figure S6: Diabetes prevalence in San Juan: county-level vs. census tract–level resolution. Comparison of average diabetes prevalence in the San Juan municipality (left panel) with high-resolution estimates by census tract (right panel). The tract-level map reveals substantial neighborhood-level variation that is not visible in aggregated county-level data. Figure S7: Relationship between diabetes prevalence and household income across PR municipalities (2024). Scatter plot of county-level diabetes prevalence versus average household income. Each point represents a municipality. A linear regression line is also shown, indicating a negative association between income and disease prevalence. References [22,28,30,31] are included in the Supplementary Materials.

## Abbreviations

The following abbreviations are used in this manuscript:

PR	Puerto Rico
CDC	Centers for Disease Control and Prevention
ADA	American Diabetes Association
HITECH	Health Information Technology for Economic and Clinical Health Act
NHANES	National Health and Nutrition Examination Survey
BRFSS	Behavioral Risk Factor Surveillance System
EHR	Electronic health record
LIS	Lab Information System
PII	Personally identifiable information
ICD-10	International Classification of Diseases (v10)
LOINC	Logical Observation Identifier Names and Codes
POI	Period of interest
DM	Diabetes mellitus
FPG	Fasting plasma glucose
GTT	Glucose tolerance test
A1C	Hemoglobin A1c
