# Supplementary material for: Chronic Disease Monitoring: Methodology for Classification Error and Self-Selection Bias Correction in Clinical Laboratory Data
Source: Healthcare (Basel). 2025 Nov 25;13(23):3056. doi: 10.3390/healthcare13233056 (PMC12692549; doi:10.3390/healthcare13233056)
Supplement: Supplementary file 1 [file healthcare-13-03056-s001.zip › healthcare-3929095-supplementary.pdf]

healthcare

MDPI

*Supplementary Information*

# Chronic Disease Monitoring: Methodology for Classification Error and Self-Selection Bias Correction in Clinical Laboratory Data

**Jesuan Betancourt [1], Efrain Betancourt [1], Abiel Roche-Lima [2] and Julian Velev [1,3,*]**

[1] Abartys Health, San Juan, PR 00907-3913, USA
[2] Center for Collaborative Research in Health Disparities, RCMI Program, Medical Science Campus, University of Puerto Rico, San Juan, PR 00936-5067, USA
[3] Department of Physics, University of Puerto Rico, San Juan, PR 00925-2537, USA
* Correspondence: julian.velev@upr.edu or jvelev@abartyshealth.com

## 1. Estimation from observational data

This work presents a methodology for correcting errors and biases in near-real-time observational data, where clinical test results are received continuously or in batches from numerous independent sources. Without control over the experimental conditions, the method relies on structural assumptions and empirical corrections. For the methodology to yield reliable and generalizable estimates, several basic preconditions must be met.

First, a large and geographically dispersed network of data sources is essential to ensure that the tested sample approximates a random sampling of the population. A small number of laboratories – or a concentration of labs in specific regions – can result in sample bias, as the data would disproportionately represent individuals living nearby. Furthermore, data from reference laboratories or those affiliated with clinics or hospitals should generally be excluded, as these facilities tend to serve a narrower, often unhealthy patient base and do not provide equal access to all population segments. In the case of Puerto Rico (PR), the dataset used in this study comes from approximately 800 independent clinical laboratories distributed across the island, offering broad and diverse population coverage.

Second, not all laboratory tests are appropriate for prevalence estimation. Tests that are ordered primarily for diagnostic purposes – especially those triggered by clinical suspicion – are prone to selection bias, overrepresenting individuals at higher risk. Additionally, expensive or invasive tests (e.g., MRI, CT scans) are not equally accessible across all socioeconomic groups. Instead, effective surveillance relies on routine screening tests that are widely administered as part of standard health checkups. Examples include the Comprehensive Metabolic Panel (CMP), Lipid Panel (LP), and Complete Blood Count (CBC) [1]. For diabetes, while hemoglobin A1c (A1C) is a common diagnostic tool, it is often ordered in response to elevated risk or suspicion of disease. In contrast, fasting plasma

 https://doi.org/10.3390/healthcare13233056

glucose (FPG), which is included in the CMP, is more likely to be administered regardless of disease status and is thus less susceptible to selection bias [2].

Third, the observation period must be sufficiently long to mitigate self-selection bias – the tendency of individuals to undergo testing at different rates based on their health status, age, or personal behavior. This process can be viewed analogously to survival analysis, where all individuals eventually undergo testing, but at different intervals. The required duration should be long enough to account for those population segments that visit healthcare providers infrequently – as healthy young individuals. Large sample sizes are also essential to reduce statistical noise within each subgroup. In this study, we focus on the year 2024 as the period of interest (POI), drawing on a dataset that spans more than five years (2020-2024) and contains tens of thousands of test results per population segment.

When these conditions are met – broad geographic sampling, equitable access to routine screening, and sufficient longitudinal coverage – observational laboratory data can support precise and unbiased estimation of disease prevalence at the population level.

## 2. Data collection

The dataset was provided by Abartys Health, a health data and analytics company operating in the PR market. The laboratory results originate from a network of approximately 800 independent clinical laboratories distributed across the island, offering extensive geographic and demographic coverage. A summary of the available glycemic test data over the whole period of intertest is presented in Table S1.

*Table S1: Sample size and glycemic test volume for the full dataset (2020-2024).*

| Sex | Persons | Results | Fasting Plasma Glucose (FPG) | Hemoglobin A1c (A1C) |
|---|---|---|---|---|
| Female | 834,987 | 4,479,242 | 3,183,900 | 1,295,342 |
| Male | 627,249 | 3,019,233 | 2,135,604 | 883,629 |
| Both | 1,462,236 | 7,498,475 | 5,319,504 | 2,178,971 |

Additionally, Figure S1 displays the number of glycemic tests performed in 2024, stratified by age group and sex. For both biomarkers and across both sexes, the number of tests increases steadily with age, reaching a peak around 60-65 years of age, after which test counts decline. This trend is consistent with greater healthcare engagement among older adults, followed by a reduction in test volume likely due to increased mortality or reduced access after retirement.

A pronounced gender disparity is also evident: women consistently undergo more glycemic testing than men across nearly all age groups, except in childhood and adolescence where the difference is minimal. This disparity can be partially attributed to

population structure – women comprise a slightly larger share of the general population beyond age 25, possibly due to gendered migration patterns. However, the larger gap observed in the lab-tested sample suggests that women are more engaged in preventive health care, leading to higher test counts independent of demographic representation.

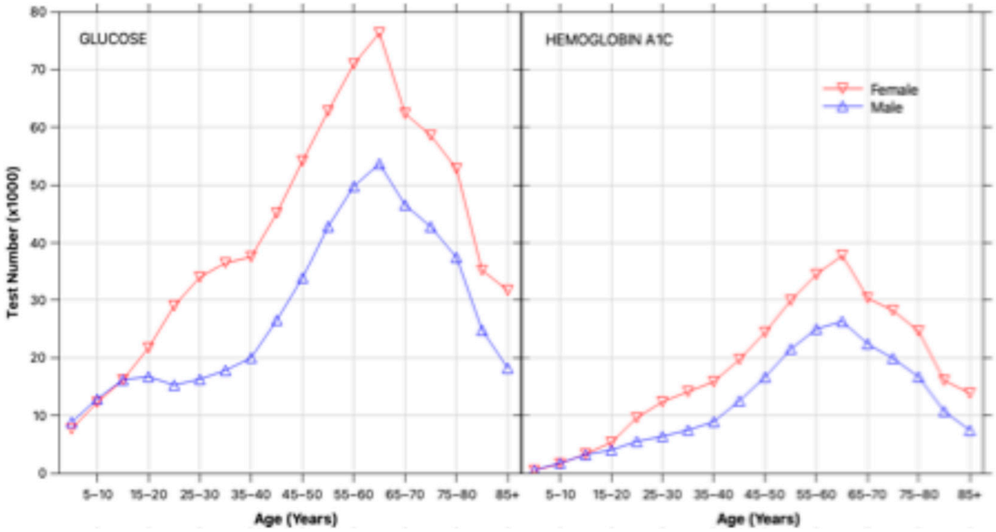


*Figure S1: Test counts by sex and age group in 2024. Number of tests performed per demographic segment for fasting plasma glucose (left panel) and hemoglobin A1c (right panel), stratified by sex and age group.*

Together, these patterns highlight the non-random nature of the tested population, reinforcing the need for bias correction methods that account for both demographic structure and healthcare-seeking behavior when estimating population-level disease prevalence.

## 3. Methodology

The use of clinical laboratory data for disease surveillance introduces inherent errors and biases. These arise not only from imperfect diagnostic performance but also from the non-random nature of the tested population. In this study, we develop a comprehensive methodology to correct for these distortions and produce an *unbiased estimator* of disease prevalence for multi-stage chronic conditions. This estimator is constructed from observable – but biased – disease frequencies, combined with empirical parameters derived from longitudinal laboratory records.

The conceptual framework is illustrated in Figure S2, which depicts both the simple binary case (disease vs. no disease) and the more general multi-stage setting. The total population is assumed to be partitioned into $M$ true disease stages, denoted $D_i$, where $D_0$ represents the absence of disease and $D_M$ the most advanced stage. However, we do not observe the true distribution directly. Instead, our data consists of a sample $S$ drawn from the population, consisting of laboratory test results classified into observed stages $D_i^*$. These observed classifications are imperfect: due to test limitations, an individual at

stage $D_j$ may be misclassified as belonging to $D_i^*$, leading to false positives or false negatives.

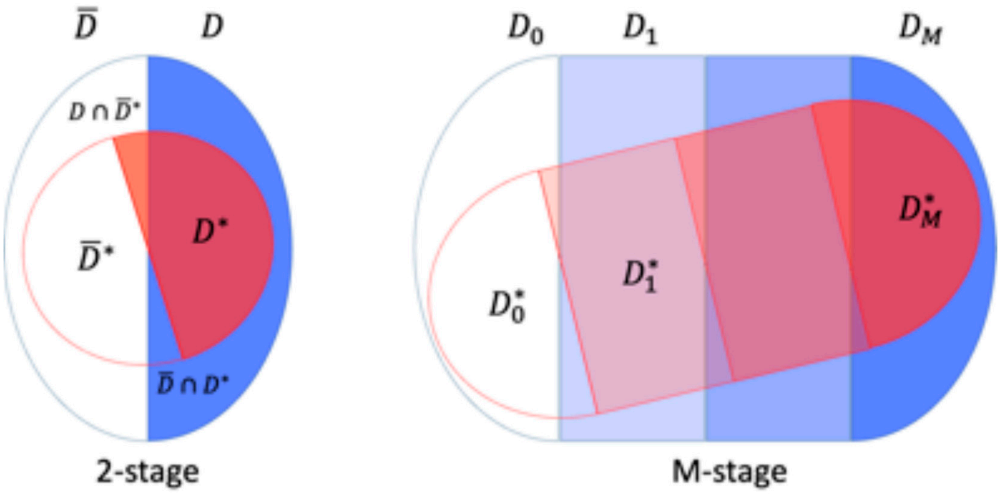


*Figure S2: Venn diagram illustrating the relationship between observed classifications in the tested sample and true disease stages in the general population, shown for a two-stage condition (left) and a multi-stage condition (right). Due to test imperfections, the observed groups ($D_i^*$) do not align with the true condition stages ($D_i$), leading to misclassification.*

From the observed data, we can immediately compute the positive frequencies of the disease stages in the tested sample as

$$\boldsymbol{F} = \left(F_{D_0}, \dots, F_{D_M}\right) = \frac{1}{N_S}\left(N_{D_0^*}, \dots, N_{D_M^*}\right)$$

where $N_{D_i^*}$ is the number of individuals classified into observed stage $D_i^*$, and $N_S$ is the total number of individuals in the sample during the POI. This frequency vector $\boldsymbol{F}$ serves as a naïve estimator of disease prevalence. However, it is systematically biased due to misclassification and self-selection effects and therefore does not accurately reflect the true distribution of disease stages in the general population.

The process of correcting these biases is illustrated in Figure S3 and detailed in the following sections. The goal is to construct an unbiased estimator

$$\boldsymbol{P} = \left(P_{D_0}, \dots, P_{D_M}\right)$$

representing the true population-level prevalence of each disease stage. This correction is performed stepwise, starting from the observed frequencies $\boldsymbol{F}$, and progressively incorporating additional empirical parameters – such as misclassification rates and stage-dependent testing probabilities – estimated from historical longitudinal data.

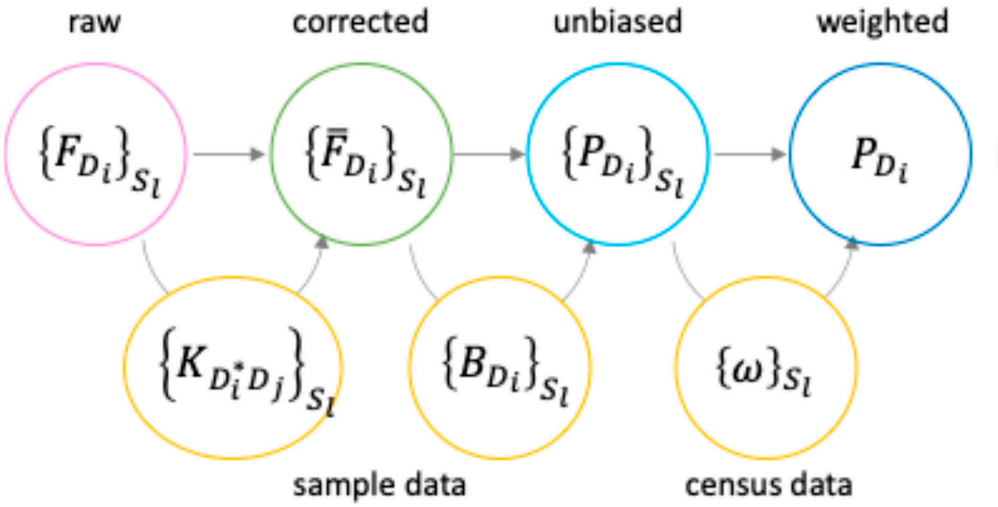


*Figure S3: Workflow for constructing the unbiased prevalence estimator.* *Starting from the observed positive frequencies $F_{D_i^*}$ for each observed disease stage $D_i^*$ and population stratum $S_l$, the unbiased estimator is built step by step using empirical parameters derived from historical data. $K_{D_i^* D_j}$ denotes the confusion matrix, which corrects for classification errors; $B_{D_j}$ represents the stage-specific testing frequency bias; and $\omega_{S_l}$ are the population weights for each stratum $S_l$.*

### *3.1. Classification errors and self-selection bias (binary case)*

First, let us assume that a condition has a binary outcome $\bar{D}$ and $D$. The goal is to estimate the unbiased prevalence of the condition $P(D)$ in the population of size $N$ based on testing a sample of the population $N_S$ with an imperfect test. The latter condition implies that the test can produce false positives $(D^* \cap \bar{D})$ or false negatives $(\bar{D}^* \cap D)$. The situation is illustrated in the Venn diagram in Figure S2.

By performing the test, we observe the raw, biased positive frequencies of the condition

$$F(D^*) \equiv F_{D^*} = \frac{N_{D^*}}{N_S} = \frac{\frac{N_{D^*}}{N}}{\frac{N_S}{N}} \cong \frac{P(D^* \cap S)}{P(S)} \equiv P(D^*|S)$$

where $P(D^* \cap S)$ is the probability of getting tested and obtaining a positive result and $P(S)$ is the probability to be tested. In other words, the positive frequencies represent the conditional probability of obtaining positive result given that the person was tested.

For an imperfect test $P(D^* \cap S) = P(D^* \cap \bar{D} \cap S) + P(D^* \cap D \cap S)$ since $\bar{D}$ and $D$ are complementary. Similarly, $P(S) = P(\bar{D} \cap S) + P(D \cap S)$. We can express the numerator via the unbiased estimators with the help of the conditional probability as follows

$$P(D^* \cap \bar{D} \cap S) = P(D^*|\bar{D} \cap S)P(\bar{D} \cap S) = P(D^*|\bar{D} \cap S)P(S|\bar{D})\big(1 - P(D)\big)$$
$$P(D^* \cap D \cap S) = P(D^*|D \cap S)P(D \cap S) = P(D^*|D \cap S)P(S|D)P(D)$$

where we have used the normalization condition $P(\bar{D}) = 1 - P(D)$. Furthermore, we can define a of vector with the rates of testing with and without the condition

$$B \equiv (P(S|\bar{D}), \quad P(S|D))$$

where the difference between these rates is the *self-selection testing bias*. These rates, and therefore the bias, are also observable. We can also define a confusion matrix

$$\boldsymbol{K} \equiv \begin{pmatrix} P(\bar{D}^*|\bar{D} \cap S) & P(\bar{D}^*|D \cap S) \\ P(D^*|\bar{D} \cap S) & P(D^*|D \cap S) \end{pmatrix} = \begin{pmatrix} S_p & 1 - S_e \\ 1 - S_p & S_e \end{pmatrix} \cong \begin{pmatrix} \frac{N_{\bar{D}^*\bar{D}}}{N_{\bar{D}}} & \frac{N_{\bar{D}^*D}}{N_D} \\ \frac{N_{D^*\bar{D}}}{N_{\bar{D}}} & \frac{N_{D^*D}}{N_D} \end{pmatrix}$$

which is the matrix of the probabilities to get a positive or negative results of the test given the presence or absence of the condition if tested. For a binary outcome, these matrix elements are related to the sensitivity $S_e$ and specificity $S_p$ of the test. The sensitivity

$$S_e = \frac{N_{D^*D}}{N_D} = \frac{N_{D^*D}}{N_{D^*D} + N_{\bar{D}^*D}} = P(D^*|D \cap S)$$

is a measure of how accurate the test is to detect the condition, and it is defined as the ratio of the true positives to the total positive cases (true positives + false negatives). Conversely, the specificity

$$S_p = \frac{N_{\bar{D}^*\bar{D}}}{N_{\bar{D}}} = \frac{N_{\bar{D}^*\bar{D}}}{N_{\bar{D}^*\bar{D}} + N_{D^*\bar{D}}} = P(\bar{D}^*|\bar{D} \cap S)$$

is a measure of how specific the test is to detect the condition, and it is defined as ratio of the true negatives to the total negative cases (true negative + false positive).

In this notation the unbiased estimator can be expressed by the biased estimators as:

$$P(D) = \frac{(F_{D^*} - K_{D^*\bar{D}})B_{\bar{D}}}{(F_{D^*} - K_{D^*\bar{D}})B_{\bar{D}} - (F_{D^*} - K_{D^*D})B_D} = \frac{F_{D^*} - (1 - S_p)}{S_e T - (1 - S_p) + F_{D^*}(1 - T)}$$

which is expressed entirely in terms of observable quantities, in particular $T = \frac{B_D}{B_{\bar{D}}}$ is the disease testing bias. The raw positive frequency is corrected everywhere for the test confusion and weighted with the testing rates in the presence or absence of the condition. In the case of no testing bias (i.e., $B_D = B_{\bar{D}}$), this expression reduces to the classic Rogan-Gladen formula [3]

In the special case of perfect tests, the confusion matrix is diagonal, and the unbiased estimator becomes:

$$P(D) = \frac{F_{D^*}B_{\bar{D}}}{B_D + F_{D^*}(B_{\bar{D}} - B_D)} = \frac{F_{D^*}}{T + F_{D^*}(1-T)}$$

and only in absence of any disease testing bias, $B_{\bar{D}} = B_D$, the positive rate is an accurate estimate of the unbiased estimate $P(D) = F_{D^*}$.

*3.2. Classification errors and self-selection bias (multi-stage case)*

The matrix notation introduced makes it easy to generalize the prevalence estimates to multi-level conditions. Let us assume that a condition has $M$ outcomes $D_0, .., D_M$ where $D_0$ stands for the absence of the condition. The goal is to estimate the unbiased vector of prevalence of the condition $P = \left(P_{D_1}, \cdots, P_{D_M}\right)$ in the population of size $N$ based on testing a sample of the population $N_S$ with an imperfect test.

Similarly, by performing the test, we observe the vector of raw, biased positive frequencies of the condition levels:

$$F \equiv \frac{1}{N_S}\left(N_{D_1^*}, \cdots, N_{D_M^*}\right) = \frac{1}{P(S)}\left(P(D_1^* \cap S), \cdots, P(D_M^* \cap S)\right)$$

where $P(D_i^* \cap S)$ is the probability of a positive result for level $i$ if tested and $P(S)$ is the probability to be tested. Each numerator takes the form $P(D_i^* \cap S) = \sum_j P\left(D_i^* \cap D_j \cap S\right)$ and the denominator $P(S) = \sum_j P\left(D_j \cap S\right)$ since $D_j$ are complementary.

Then we express the joint probabilities through the unbiased prevalence estimators and using the previously defined notation

$$P\left(D_i^* \cap D_j \cap S\right) = P\left(D_i^* | D_j \cap S\right) P\left(D_j \cap S\right) = P\left(D_i^* | D_j \cap S\right) P\left(S | D_j\right) P\left(D_j\right) = K_{D_i^* D_j} B_{D_j} P_{D_j}$$
$$P\left(D_j \cap S\right) = P\left(S | D_j\right) P\left(D_j\right) = B_{D_j} P_{D_j}$$

Substituting in the expression for the positive frequencies we can show that $P$ is the solution of the linear system of equations

$$\sum_j \left(F_{D_i^*} - K_{D_i^* D_j}\right) B_{D_j} P_j = 0$$

where the testing rate and confusion matrix are straightforward generalizations

$$B \equiv \left(P(S|D_0) \quad \cdots \quad P(S|D_M)\right)$$

$$K \equiv \begin{pmatrix} P(D_0^* | D_0 \cap S) & \cdots & P(D_0^* | D_M \cap S) \\ \vdots & \ddots & \vdots \\ P(D_M^* | D_M \cap S) & \cdots & P(D_M^* | D_M \cap S) \end{pmatrix} \cong \begin{pmatrix} \frac{N_{D_0^* D_0}}{N_{D_0}} & \cdots & \frac{N_{D_0^* D_M}}{N_{D_M}} \\ \vdots & \ddots & \vdots \\ \frac{N_{D_M^* D_0}}{N_{D_0}} & \cdots & \frac{N_{D_M^* D_M}}{N_{D_M}} \end{pmatrix}$$

and the $N_{D_i}$'s are the total number of cases $D_i$ including true positives and false negatives. Therefore, the coefficients $A_{ij} = \left(F_{D_i^*} - K_{D_i^* D_j}\right) B_{D_j}$ has the meaning of the level positive frequencies corrected for the test confusion and weighted by the disease testing rates as before.

A particular kind of classification error comes from the use of medications specifically designed to control a condition. The use of these medications reduces the sensitivity of the test by increasing the number of false negatives. For otherwise perfect tests, a person with $D_i$ stage of the condition without medication will have $P(D_i^* | D_i \cap S) = 1$ and $P(D_j^* | D_i \cap S)\big|_{j<i} = 0$. The use of medication would result in $\tilde{P}(D_i^* | D_i \cap S) < 1$ and $\tilde{P}(D_j^* | D_i \cap S)\big|_{j<i} > 0$ as $D_i$ appearing as $D_{j<i}^*$ in the results. This results in an upper diagonal confusion matrix

$$\widetilde{K} = \begin{pmatrix} \tilde{P}(D_0^* | D_0 \cap S) & \cdots & \tilde{P}(D_0^* | D_M \cap S) \\ \vdots & \ddots & \vdots \\ 0 & \cdots & \tilde{P}(D_M^* | D_M \cap S) \end{pmatrix}$$

Furthermore, we car rearrange the equation

$$\sum_j \left(\bar{F}_{D_k^*} - \delta_{kj}\right) B_{D_j} P_j = 0$$

where $\bar{F}_{D_k^*} = \sum_i K_{D_k D_i^*}^{-1} F_{D_i^*}$ is the positive rate corrected for the test precision error. The unbiased estimator $\boldsymbol{P}$ can be obtained by solving the system of equations above with the normalization condition $|\boldsymbol{P}| = 1$.

Thus the bias and error correction can be done in the following steps: $\boldsymbol{F}_l, \boldsymbol{B}_l, \boldsymbol{K}_l, \boldsymbol{\omega}_l \rightarrow \bar{\boldsymbol{F}}_l, \boldsymbol{B}_l, \boldsymbol{\omega}_l \rightarrow \boldsymbol{P}_l, \boldsymbol{\omega}_l \rightarrow \boldsymbol{P}$ where all the quantities on the initial step are estimated from empirical data. It is easy to show that for $M = 2$ we obtain the result for the binary outcome case.

### *3.3. Demographic reweighting*

A more refined correction of the self-selection bias can be performed by separating the population in strata $S_l(\alpha, \gamma, ..)$ defined by a set of parameters such as age ($\alpha$), gender ($\gamma$), etc. The stratification parameters are only limited by the availability of these indicators in the test data and the ability to obtain independent population weights $\omega_l$ for these strata in the general population. Then, the unbiased estimator can be obtained from a weighted sum of the strata prevalence

$$P(D_i) = \sum_l P(D_i \cap S_l) = \sum_l P(S_l) P(D_i | S_l) = \sum_l \omega_l \, P(D_i | S_l)$$

where the weights of the strata are replaced with the population weights $\omega_l = \frac{N_{S_l}}{N}$, rather than the sample weights $\omega_l^* = \frac{N_{S_l}^*}{N_S}$.

Stable estimation of the confusion and self-selection matrices requires sufficient sample size within each demographic and geographic stratum. Using the variance of a binomial proportion as a guide

$$\mathrm{Var}(\hat{p}) = \frac{p(1-p)}{n}$$

several thousand observations per stratum ensure sampling error below ~1 percentage point for typical prevalence ranges (10–20%).

## 3. Prevalence estimation

While diabetes prevalence estimates are presented in the main paper (Figure 3), Figure S4 shows the estimated prevalence of prediabetes by population segment for 2024. The overall trends closely mirror those observed for diabetes: prevalence increases steadily with age and is consistently higher in males than in females across most age groups.

However, the correction steps have a distinct effect on prediabetes estimates. In particular, the classification error correction tends to reduce the raw positive frequencies $F(D_1)$ for prediabetes. This is because a subset of individuals whose test results fall within the prediabetic range are, in fact, diabetic patients managing their condition with insulin, which artificially lowers their glycemic values. As in the diabetes case, the self-selection bias correction also reduces the prevalence estimate, accounting for the fact that individuals with known or suspected conditions are more likely to undergo testing.

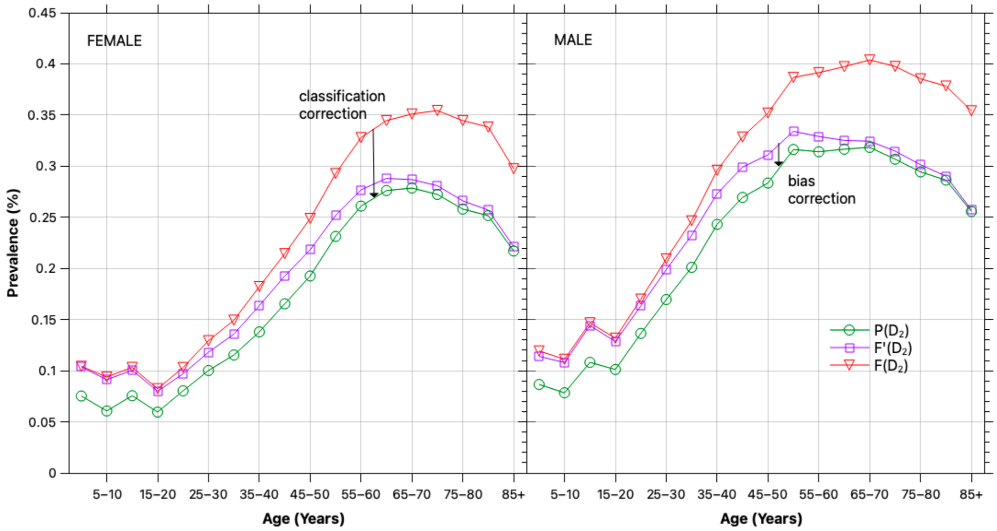


*Figure S4: Prediabetes prevalence by sex and age group in Puerto Rico in 2024. Estimated prevalence of prediabetes across demographic segments, stratified by sex and age group. Results reflect corrected estimates after adjustment for classification error and self-selection bias.*

Historical prevalence estimates for diabetes and prediabetes are presented in Table S2. The data indicate that both diabetes and prediabetes prevalence have remained relatively stable over the past five years.

*Table S2: Historical prevalence of diabetes and prediabetes in Puerto Rico (2020–2024). Yearly estimates of diabetes and prediabetes prevalence among adults, based on laboratory data corrected for classification error and self-selection bias.*

| Year | Diabetes (%) | Prediabetes (%) |
|---|---|---|
| 2020 | 18.8 | 22.3 |
| 2021 | 19.9 | 24.3 |
| 2022 | 19.7 | 23.8 |
| 2023 | 18.9 | 23.9 |
| 2024 | 18.0 | 22.8 |
| 2020 | 18.8 | 22.3 |

## 4. Socio-demographic analysis

By mapping patients' counties based on the city field in their addresses, we computed county-level prevalence across PR. The results are visualized in Figure S5, which shows substantial variation in diabetes prevalence across municipalities.

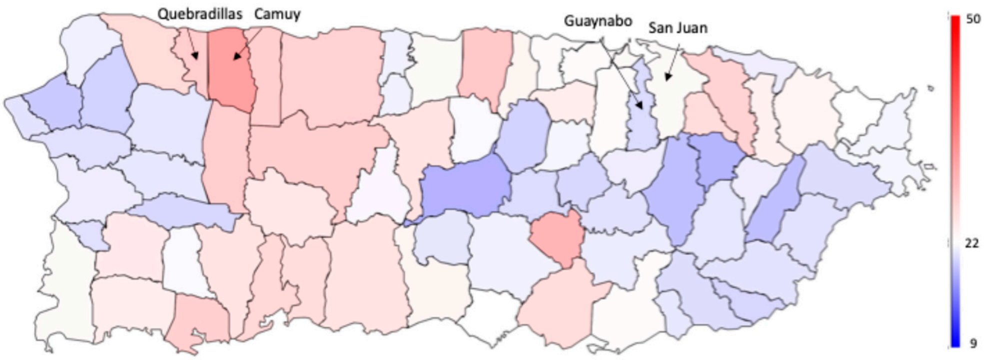


*Figure S5: Diabetes prevalence in Puerto Rico by county in 2024.*

A clear inverse relationship emerges between average household income and diabetes prevalence. Counties with lower average income – such as Quebradillas (29.6%) and Camuy (36.5%) – exhibit significantly higher rates of diabetes compared to wealthier municipalities like Guaynabo (19.2%) and San Juan (22.3%). This pattern is consistent with established evidence linking socioeconomic disadvantage to chronic disease burden and highlights the need for geographically targeted prevention and intervention strategies.

The large volume of laboratory data also enables high-resolution mapping of diabetes prevalence at the sub-county level. By geocoding patient addresses, we assign individuals to specific census tracts, allowing for more granular spatial analysis. As shown in Figure S6, this dramatically enhances the geographic resolution compared to county-level estimates.

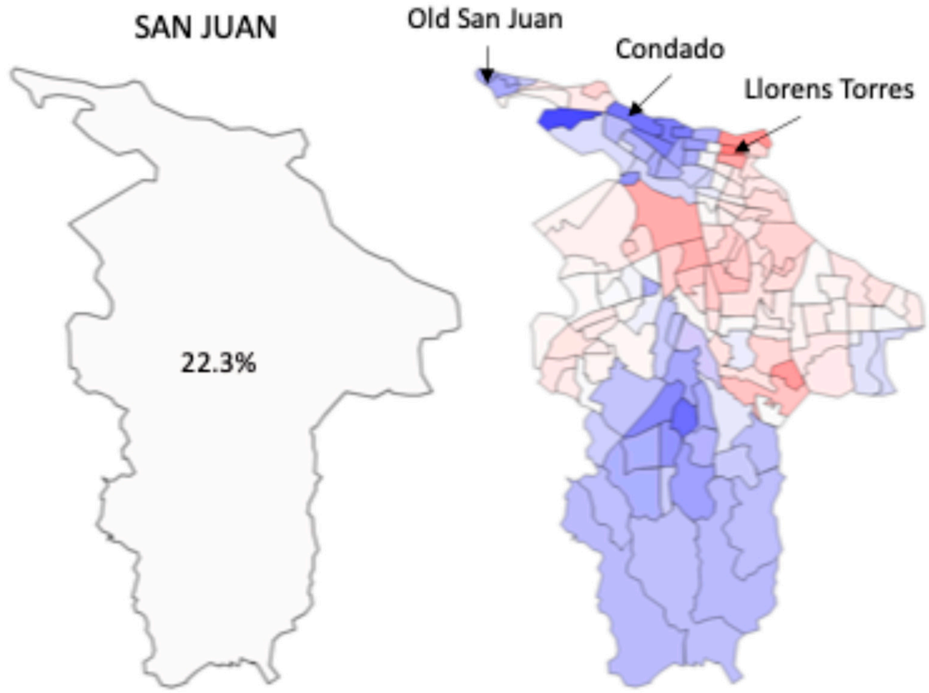


*Figure S6: Diabetes prevalence in San Juan: county-level vs. census tract–level resolution. Comparison of average diabetes prevalence in the San Juan municipality (left panel) with high-resolution estimates by census tract (right panel). The tract-level map reveals substantial neighborhood-level variation that is not visible in aggregated county-level data.*

For example, while the average prevalence in the broader San Juan municipality is approximately 22%, the census tract map reveals significant variation across neighborhoods. Wealthier areas such as Old San Juan and Condado exhibit substantially lower prevalence rates, whereas lower-income neighborhoods, including the government-sponsored housing complex Llorens Torres, display markedly higher rates. This fine-scale resolution highlights within-county disparities that would otherwise be obscured and offers a powerful tool for targeting public health interventions at the community level.

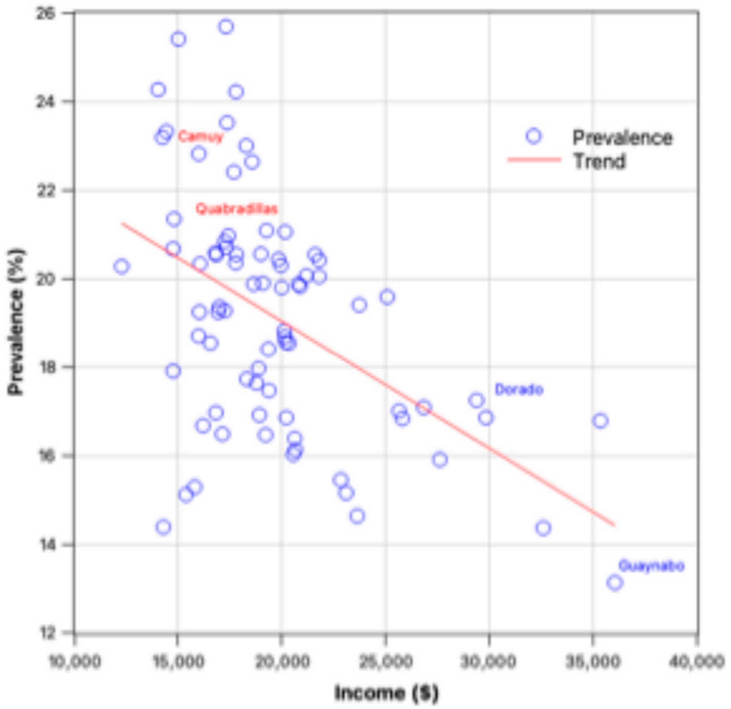


*Figure S7:* ***Relationship between diabetes prevalence and household income across PR municipalities (2024)****. Scatter plot of county-level diabetes prevalence versus average household income. Each point represents a municipality. A linear regression line is also shown, indicating a negative association between income and disease prevalence.*

To quantify the relationship between socioeconomic status and diabetes burden, we linked the 2024 diabetes prevalence estimates with the most recent Social Determinants of Health (SDOH) dataset [4]. We performed an ordinary least squares (OLS) regression of diabetes prevalence against median household income using county-level data for 2024, the results of which is shown in Figure S7. The analysis reveals a significant linear trend: diabetes prevalence decreases as average household income increases. The estimated slope is –2.9 ± 0.6 percentage points per $10,000 increase in income, with a coefficient of determination of $R^2 = 24.4\%$. In other words, for every additional $10,000 in average household income, the diabetes prevalence rate decreases by approximately 3%. Income alone accounts for about one-quarter of the observed variation in prevalence across municipalities.

The relatively modest $R^2$ reflects the substantial variance in diabetes prevalence among lower-income counties. One possible explanation is the low degree of residential segregation in PR, where households of varying socioeconomic status often live in close geographic proximity. This spatial mixing may attenuate income gradients at the county level and suggests that finer-resolution analysis – such as at the census tract level – may reveal stronger local associations.

## Abbreviations

The following abbreviations are used in this manuscript:

| | |
|---|---|
| PR | Puerto Rico |
| POI | Period of interest |
| DM | Diabetes mellitus |
| FPG | Fasting plasma glucose |
| A1C | Hemoglobin A1c |

## References

1. Pagana, K.D.; Pagana, T.J. *Laboratory Tests and Diagnostic Procedures*; 5th ed.; Mosby: St. Louis, MO, 2012; ISBN 9780323057479.
2. loinc.org Comprehensive Metabolic 2000 Panel - Serum or Plasma.
3. Rogan, W.J.; Gladen, B. Estimating Prevalence from the Results of a Screening Test. *Am J Epidemiol* **1978**, *107*, 71–76, doi:10.1093/oxfordjournals.aje.a112510.
4. Agency for Healthcare Research and Quality Social Determinants of Health Database.
